# Dopamine D1/D5 Receptors in the Retrosplenial Cortex Are Necessary to Consolidate Object Recognition Memory

**DOI:** 10.3389/fnbeh.2022.922971

**Published:** 2022-07-07

**Authors:** Ana Belén de Landeta, Jorge H. Medina, Cynthia Katche

**Affiliations:** ^1^CONICET-Universidad de Buenos Aires, Instituto de Biología Celular y Neurociencia “Prof. E. De Robertis” (IBCN), Buenos Aires, Argentina; ^2^Universidad de Buenos Aires, Facultad de Medicina, Buenos Aires, Argentina; ^3^Instituto Tecnológico de Buenos Aires (ITBA), Buenos Aires, Argentina

**Keywords:** dopamine, long-term memory, SCH23390, SKF38393, posterior cingulate cortex

## Abstract

The retrosplenial cortex (RSC) has been widely related to spatial and contextual memory. However, we recently demonstrated that the anterior part of the RSC (aRSC) is required for object recognition (OR) memory consolidation. In this study, we aimed to analyze the requirement of dopaminergic inputs into the aRSC for OR memory consolidation in male rats. We observed amnesia at 24-h long-term memory when we infused SCH23390, a D1/D5 dopamine receptors antagonist, into aRSC immediately after OR training session. However, the same infusion had no effect on OR short-term memory. Then, we analyzed whether the ventral tegmental area (VTA) is necessary for OR consolidation. VTA inactivation by intra-VTA administration of muscimol, a GABA_A_ agonist, immediately after an OR training session induced amnesia when animals were tested at 24 h. Moreover, we observed that this VTA inactivation-induced amnesia was reversed by the simultaneous intra-aRSC delivery of SKF38393, a D1/D5 receptor agonist. Altogether, our results suggest that VTA dopaminergic inputs to aRSC play an important modulatory role in OR memory consolidation.

## Introduction

Recognition memory refers to the recall and awareness of a familiar event, individual, item, or place, allowing animals to discriminate between novel and familiar stimuli. In particular, the object recognition (OR) task has been widely used for studying the “what” component of recognition memory. The anterior retrosplenial cortex (aRSC) was recently observed to participate in OR memory consolidation (de Landeta et al., 2020), i.e., the storage of the “what” component of recognition memory. Nevertheless, there is much to unravel about the mechanisms involved in the OR memory consolidation process.

Understanding the mechanisms involved in memory consolidation is a main topic in memory research, which is relevant to better understand some memory disorders and to analyze molecular targets related to those disorders. In particular, exposure to novel stimuli induces dopamine release from the ventral tegmental area (VTA) into the hippocampus to form long-term memory (LTM) (Lisman and Grace, 2005). Moreover, dopamine is known to regulate OR memory in the prefrontal and perirhinal cortices (Nagai et al., 2007; Balderas et al., 2013; De Bundel et al., 2013; Rossato et al., 2013). Thus, dopamine is a strong candidate for modulating OR memory consolidation in the RSC.

In this regard, the RSC receives dopaminergic projections from the VTA (Berger et al., 1985; Oades and Halliday, 1987), a structure that consists mainly of dopaminergic neurons (Morales and Margolis, 2017) and that was observed to be necessary for OR memory consolidation (Rossato et al., 2013). In addition, the RSC expresses D1/D5 receptors (Diop et al., 1988) and D1/D5 activity in the aRSC is necessary and sufficient to form a long-lasting aversive memory (Katche et al., 2013). In this scenario, we hypothesized that dopaminergic inputs from VTA to aRSC are essential for OR memory consolidation. Here, we combined pharmacological and behavioral approaches to assess the role of the dopaminergic tone in the aRSC during OR memory consolidation.

## Methods

### Subjects

We used a total of 92 2.5-month-old male Wistar rats (Instituto de Biología Celular y Neurociencia, CONICET-UBA) weighing about 220–300 g. Animals were housed in groups of three per cage and maintained under a 12 h light/dark cycle (lights on at 7:00 a.m.) at 21–23°C with water and food *ad libitum*. Experimental procedures followed the guidelines of the US National Institutes of Health (NIH) Guide for the Care and Use of Laboratory Animals and were approved by the Animal Care and Use Committee at the University of Buenos Aires (CICUAL).

### Surgery

Rats were implanted bilaterally under deep ketamine/xylazine anesthesia (40 and 2 mg/kg, respectively) with a 1-cm 22 G guide cannula in the aRSC at AP −3.9, L ±0.5, DV −1.8, and VTA at AP −5.3, L ±1.0, and DV −7.2, coordinates in mm from Bregma according to the atlas of Paxinos and Watson (Paxinos and Watson, 2007). Cannulas were fixed to the skull with dental acrylic. Obturators were then inserted into the cannula to prevent blockage. After 4 or 5 days of recovery from surgery, the animals were handled gently once a day for 2 days and then trained in the OR task.

### Drug Infusion

To study the dopaminergic input we infused into the aRSC, the D1/D5 dopamine receptor antagonist SCH23390 hydrochloride (Sigma Aldrich, Germany) and the agonist SKF38393 hydrochloride (Sigma Aldrich, Germany) at a dose of 0.75 μg per side and 12.5 μg per side, respectively. We infused the GABA_A_ receptor agonist muscimol (Sigma Aldrich, Germany) at a dose of 0.1 μg per side into the VTA immediately after the training session to study memory consolidation.

All drugs except SKF38393 were dissolved in sterile saline; SKF38393 was dissolved in 10% DMSO and sterile saline. Solutions used for dissolving the drugs were infused in the control group of the experiments (Vehicle, Veh). For all drugs infused, the entire infusion procedure took around 4 min, and the infusion rate was 1 μl/min. Infusions into the aRSC were 1 μl/side, while those in the VTA were of 0.5 μl/side. Injector needles were 0.1 and 0.15 cm longer than the cannula for aRSC and VTA, respectively. Injectors were left in place for an additional minute following infusion before they were removed carefully to avoid backflow.

### Cannula Placement

**Figure 1:**
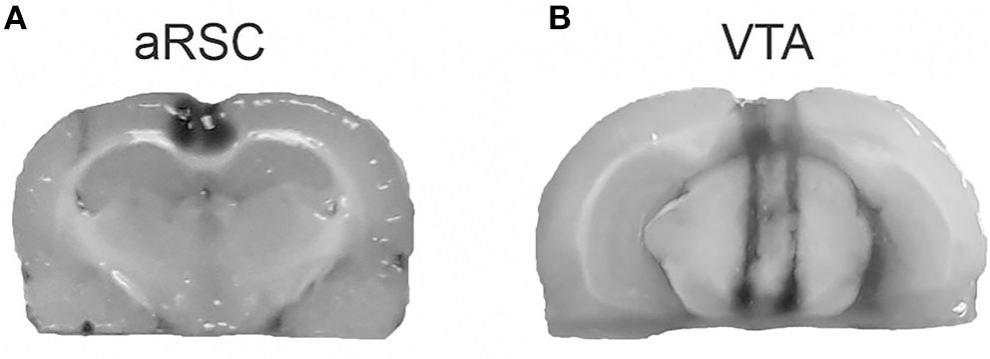
Representation of the infusion area. Pictures show the methylene blue infusions area (black) for aRSC **(A)** and VTA **(B)**.

Cannula placement was verified after the end of the behavioral procedures by infusions of 1 μl into the aRSC (Figure 1A) or 0.5 μl into the VTA (Figure 1B) of 4% methylene blue in saline. A histological examination of cannula placements was performed. Only the behavioral data from animals with the cannula located in the intended site were included in the final analysis (20 animals were excluded from the analysis).

### Y-Shape Object Recognition

We performed the OR task as previously described (de Landeta et al., 2020, 2021). In brief, we habituated the animals to the empty Y-maze for 10 min, and the following day we trained the animals with two identical objects for 5 min. We then test memory 3 or 24 h after training; during the test session, we let the animals explore one object from the training session (familiar object) and one novel object for 3 min. The novel object or its position were selected by chance and were counterbalanced between animals. Objects were made of glass, metal, or plastic. The objects and apparatus were cleaned with a solution of soap, alcohol, and water before being presented to each animal.

In both training and test sessions, we used manual timers to score the time, the rodent spent exploring the objects (sniffing or touching while sniffing or facing the object). We calculated the novel object discrimination index as the exploration time of the novel object minus the exploration time of the familiar object divided by the total exploration time. Indexes significantly greater than zero were indicators of memory. We analyzed data from animals that had a minimum exploration time of 15 s/per object during the training session showing no preference for any of the sampled objects (<65% of preference for one object during training session) and that explored more than 15 s during the test (nine animals were excluded from the analysis). Total exploration times for each experiment and manipulation are shown in Table 1.

**Table 1:** Total training and test sessions' exploration times for each manipulation.

**Figure**	**Group**	**Training**		**Test**		**dF**
		**Expl**	***p*-value**	**Expl**	***p*-value**	
		**time (s)**		**time (s)**		
2A			0.77		0.89	12
	Veh	63.1 ± 17.2		42.7 ± 17.2		
	SCH	65.6 ± 16.0		41.4 ± 12.2		
2B			0.35		0.05	13
	Veh	73.5 ± 16.6		44.2 ± 15.2		
	SCH	84.7 ± 28.0		27.7 ± 14.6		
3A			0.12		0.16	19
	Veh	71.6 ± 12.9		41.3 ± 15.8		
	Mus	83.0 ± 19.5		32.2 ± 11.0		
3B			0.27		0.42	37
	Veh-Veh	100.4 ± 19.5		40.4 ± 16.5		
	Veh-Musc	95.7 ± 12.4		35.3 ± 16.8		
	SKF-Veh	98.3 ± 23.9		47.5 ± 18.6		
	SKF-Musc	83.9 ± 24.3		39.0 ± 13.6		

*Mean ± SD exploration time for each experiment during training and test sessions. Results of two-tailed Student's t-test or ANOVA for the exploration time in each experiment*.

### Data Analysis

As we used a between-subjects design for our experiments, behavioral data were analyzed using the unpaired Studen's *t*-test between groups or the theoretical value 0 and the two-way ANOVA. We checked the normality of data using the Shapiro–Wilk test. We used Graph Pad Prism 8 (Graphpad, USA) for statistical analysis. For all analyses, the α level was set at 0.05 and the statistical power at 90% (G^*^Power, Universität Düsseldorf). All data are presented as mean ± SEM.

## Results

**Figure 2:**
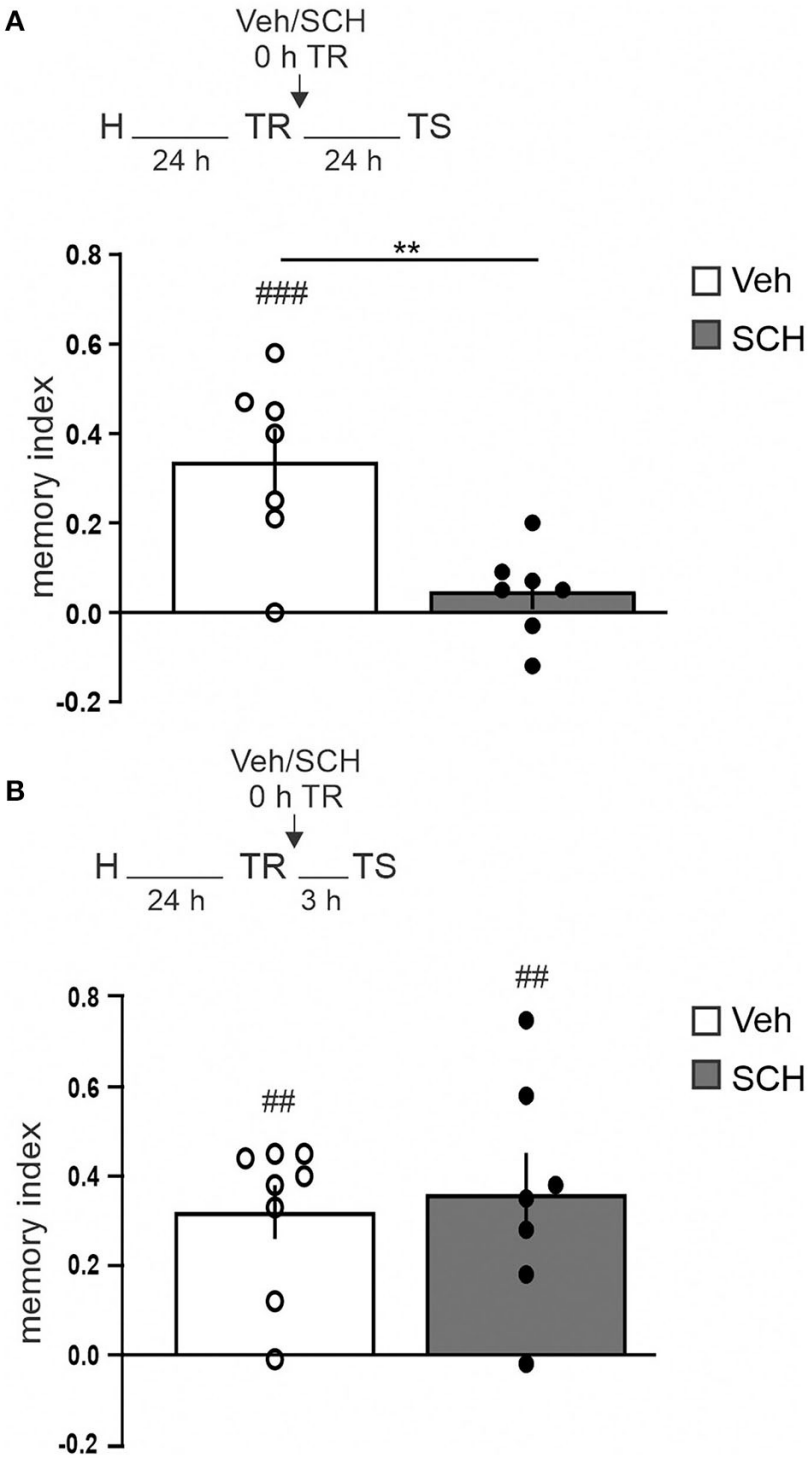
aRSC requires D1/D5 activity for object recognition memory consolidation. Saline (Vehicle, Veh, white bar) or D1/D5 antagonist (SCH23390, SCH, gray bar) was infused into aRSC immediately after training. Graphics show the discrimination index from animals tested **(A)** 24 h or **(B)** 3 h after the training session. Data are expressed as mean ± SEM. **p < 0.01, Veh vs. SCH, two-tailed Student's *t*-test; ^*##*^*p* < 0.01, ^*###*^*p* < 0.001, Group vs. 0, two-tailed Student's *t*-test. **(A)** *n* = 7, **(B)** *n* = 7–8.

To analyze the requirement of the dopaminergic input for OR memory consolidation in the aRSC, we infused SCH23390 (0.75 μg/side, D1/D5 receptors antagonist) into the aRSC immediately after the training session and tested 3 h after for short-term memory (STM) or 24 h for LTM. We observed a clear-cut amnesia at 24 h in animals infused with SCH23390, while the control group had intact memory (Figure 2A, Studen's *t*-test. SCH vs. Vehicle: *p* = 0.0042, *t* = 3.522, *df* = 12. SCH vs. 0: *p* = 0.2842, *t* = 1.176, *df* = 6. Veh vs. 0: *p* = 0.0039, *t* = 4.549, *df* = 6. *n*_SCH_ = 7, *n*_Veh_ = 7). However, we did not observe differences in the exploration pattern between control and SCH-infused animals when testing STM, showing both groups preference for the novel object (Figure 2B, Studen's *t*-test. Veh vs. SCH: *p* = 0.7419, *t* = 0.3365, *df* = 13. SCH vs. 0: *p* = 0.0097, *t* = 3.734, *df* = 6. Veh vs. 0: *p* = 0.0012, *t* = 5.261, *df* = 7. *n*_SCH_ = 7, *n*_Veh_ = 8). These results show that blocking dopaminergic signaling in the aRSC prevents OR memory consolidation but not initial formation.

**Figure 3:**
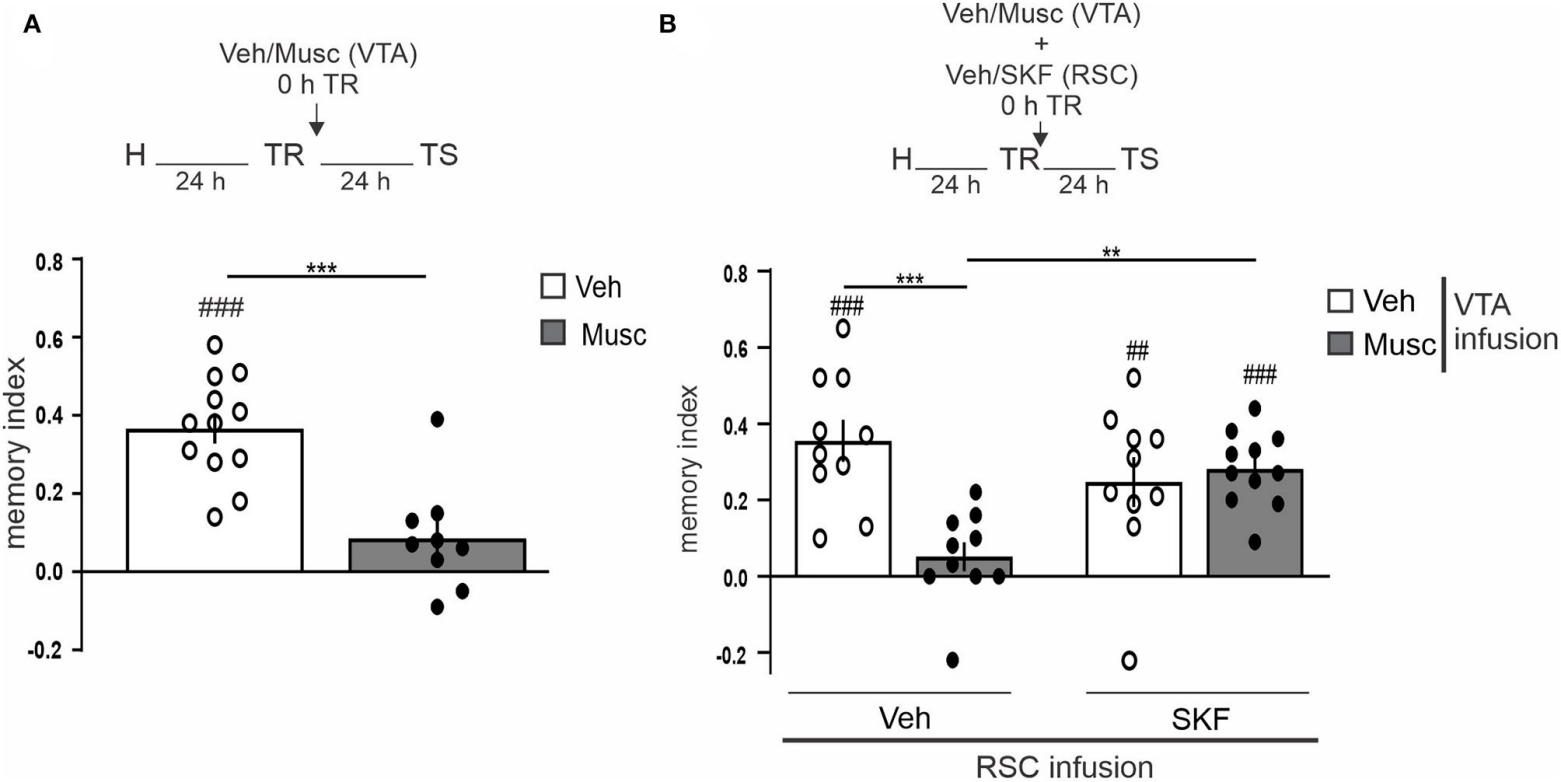
VTA dopaminergic input to aRSC is required for object recognition memory consolidation. **(A)** Saline (Vehicle, Veh, white bar) or GABA_A_ agonist (Muscimol, Musc, gray bar) infusions were made into VTA immediately after the training session; animals were tested 24 h after the training. **(B)** simultaneously, saline (Vehicle, Veh, white bar) or GABA_A_ agonist (Muscimol, Musc, gray bar) was infused into VTA and 10% DMSO (Vehicle, Veh) or D1/D5 agonist (SKF38393, SKF) was infused into aRSC immediately after the training. Test session was performed 24 h after training. Data are expressed as mean discrimination index ± SEM. **(A)** ***p < 0.001, Veh vs. Musc, two-tailed Student's *t*-test. **(B)** ***p < 0.001, **p < 0.01, Tukey's multiple comparison test after two-way ANOVA (only biologically relevant comparisons are shown). **(A,B)** ^*###*^*p* < 0.001, ^*##*^*p* < 0.01, Group vs. 0, two-tailed Student's *t*-test. **(A)** *n* = 9–12, **(B)** *n* = 10–11.

Next, we decided to study the possible involvement of the VTA in the dopaminergic modulation of OR LTM in aRSC. We transiently inactivated the VTA by infusing muscimol (0.1 μg/side, GABA_A_ agonist) immediately after the training session and tested 24 h LTM. The VTA-inactivated group did not show memory, while the control group showed preference for the novel object (Figure 3A, Studen's *t*-test. *p* = 0.0001, *t* = 4.722, *df* = 19; Musc vs. Veh: *p* = 0.0996, *t* = 1.862, *df* = 8; Musc vs. 0: *p* < 0.0001, *t* = 9.558, *df* = 11; Veh vs. 0: *n*_Musc_ = 9, *n*_Veh_ = 12). This result indicates that VTA is required for OR memory consolidation, and it is consistent with previous results using another OR task (Rossato et al., 2013). Thus, we then studied whether this amnesia could be prevented by mimicking the dopamine input in the aRSC. We observed that the co-infusion of SKF38393 (12.5 μg/side, D1/D5 receptors agonist) into the aRSC immediately after the training session reversed the amnesic effect of muscimol-induced VTA inactivation (Figure 3B, p_interaction_ = 0.0012, *F*_interaction_ = 12.23, Tukey's multiple comparisons test after two-way ANOVA_(1, 37)_ factors: infusion into VTA and infusion into aRSC. Studen's *t*-test: *p* = 0.0001, *t* = 6.474, *df* = 9; Veh–Veh vs. 0: *p* = 0.0044, *t* = 3.768, *df* = 9; Veh–SKF vs. 0: *p* = 0.2177, *t* = 1.325, *df* = 9; Musc–Veh vs. 0: *p* < 0.0001, *t* = 9.453, *df* = 10; Musc–SKF vs. 0: n_Veh−*Veh*_ = 10, n_Veh−*SKF*_ = 10, n_Musc−*Veh*_ = 10, n_Musc−*SKF*_ = 11). This result suggests that dopamine from VTA is not only necessary but also sufficient for OR memory consolidation in aRSC.

## Discussion

Our results suggest that the dopaminergic input from the VTA to the aRSC is necessary for modulating long-term OR memory consolidation. The results shown here are in line with others that showed the modulation of the dopaminergic system in OR memory by observing the enhancement of LTM when using systemic injections of dopamine D1/D5 receptor agonist SKF38393 (de Lima et al., 2011) or inhibiting the catechol-O-methyltransferase (Detrait et al., 2016). Moreover, infusion of the D1/D5 antagonist SCH23390 into the perirhinal cortex (Balderas et al., 2013), hippocampus (De Bundel et al., 2013; Furini et al., 2014; Neves et al., 2020, but see Rossato et al., 2013), amygdala (Rossato et al., 2013), or prefrontal cortex (Nagai et al., 2007; De Bundel et al., 2013; Rossato et al., 2013) produced 24 h OR amnesia, like our result when infusing SCH23390 into the aRSC. In addition, blocking dopamine reuptake in the insular cortex of an Alzheimer's disease mice model reversed the STM and LTM object amnesia in those mice (Guzmán-Ramos et al., 2012). Moreover, hippocampal dopaminergic tone is essential for object memory persistence (Neves et al., 2020; Vargas et al., 2020; Lima et al., 2022) and reconsolidation (Rossato et al., 2015; Gonzalez et al., 2021).

On the contrary, SCH23390 failed to disrupt STM formation in the medial prefrontal cortex, perirhinal cortex, and hippocampus (Savalli et al., 2015). Despite this, another study showed that the inhibition of dopaminergic activity by SCH23390 systemic administration or its infusion into the prelimbic cortex impaired OR STM (Clausen et al., 2011). Inconsistency between the results shown in these studies could be related to methodological differences, such as drug concentration and the strain of rats used. In particular, we did not find an effect of SCH23390 infusion into aRSC when testing STM. We suggest that the discrepancy between Clausen's study and ours might be due to differences in the infusion time points and in the neocortical area analyzed. In our study, we prefer to infuse SCH23390 immediately after training rather than before training; in this way, we could check whether the effect of SCH23390 on LTM was due to dopamine requirements for memory consolidation (i.e., memory stabilization) rather than deficits in acquisition or initial formation.

Although our study and others showed OR LTM impairment by SCH23390, we cannot exclude that part of this effect might be due to SCH23390 agonist activity on serotonin 5-HT_2C_ receptors (Millan et al., 2001). Nevertheless, it was observed that i.p. administration of a 5-HT_2C_ agonist, compound (+)-22a, improved OR LTM in a schizophrenia model (NR1-KD mice) (Cheng et al., 2016). Also, i.p. administration of the 5-HT_2C_ antagonist, RO 60-0491, enables OR LTM formation in animals that do not show LTM (Pitsikas and Sakellaridis, 2005), and the non-specific 5-HT_2C_ antagonist, agomelatine, improved the OR memory of stressed mice (Gumuslu et al., 2014), though blocking of 5-HT_2C_ receptors reinforces frontocortical dopaminergic transmission (Millan et al., 2003). Thus, 5-HT_2C_ receptor activity could be related to OR memory formation improvement. This strengthens that our results are related to SCH23390 activity over D1/D5 receptors.

The requirement of VTA for OR memory consolidation observed in this study is similar to that previously shown in another OR task (Rossato et al., 2013). In addition, our results showed that mimicking dopamine input by the simultaneous infusion of a D1/D5 agonist into the aRSC prevented the amnesia produced by VTA inactivation. Likewise, D1/D5 activity in the medial prefrontal cortex and amygdala together, but not each structure alone, could prevent the effect of VTA inactivation (Rossato et al., 2013). The main difference between Rossato's work and ours is that we observed that local SKF38393 only in the aRSC prevents the VTA inactivation effect, suggesting that the aRSC is a prime structure for OR processing. Considering its functional connectivity with many brain regions of the OR network (de Landeta et al., 2021), we suggest that aRSC could be relevant for receiving and sending information about different features of the objects, orchestrating object memory consolidation. However, when the aRSC is not properly functioning during memory acquisition, this role might be taken over by other brain structures (de Landeta et al., 2020).

Our results showed for the first time that dopamine is required in the aRSC for OR memory consolidation; we demonstrated that dopamine is both necessary and sufficient to consolidate OR memory in the aRSC. These results also suggest the involvement of VTA inputs to the OR memory network for the proper memory consolidation. Considering VTA cellular diversity and the existence of neurons that co-release dopamine and either GABA or glutamate (Morales and Margolis, 2017), we cannot conclude about the nature of VTA inputs into the aRSC and their effect on OR memory. To consolidate the link between the effect of VTA transient inactivation and D1/D5 signaling in the aRSC, further experiments are needed to selectively manipulate the VTA dopaminergic neurons projecting to the aRSC.

## Data Availability Statement

The raw data supporting the conclusions of this article will be made available by the authors, without undue reservation.

## Ethics Statement

The animal study was reviewed and approved by Animal Care and Use Committee of the University Buenos Aires (CICUAL). School of Medicine, University of Buenos Aires.

## Author Contributions

ABL, JHM, and CK designed the experiments and wrote the manuscript. CK led the research. ABL performed the experiments and analyzed the data. All authors contributed to the article and approved the submitted version.

## Funding

This study was supported by a grant from the National Agency of Scientific and Technological Promotion of Argentina (ANPCyT, Argentina, grant number 2018-00762), the Young IBRO Maternity/Parenthood Grant to CK and the National Scientific and Technical Research Council (CONICET, Argentina).

## Conflict of Interest

The authors declare that the research was conducted in the absence of any commercial or financial relationships that could be construed as a potential conflict of interest.

## Publisher's Note

All claims expressed in this article are solely those of the authors and do not necessarily represent those of their affiliated organizations, or those of the publisher, the editors and the reviewers. Any product that may be evaluated in this article, or claim that may be made by its manufacturer, is not guaranteed or endorsed by the publisher.

## References

[B1] Balderas I. Moreno-Castilla P. Bermudez-Rattoni F. (2013). Dopamine D1 receptor activity modulates object recognition memory consolidation in the perirhinal cortex but not in the hippocampus. Hippocampus 23, 873–878. 10.1002/hipo.22143 23674387

[B2] Berger B. Verney C. Alvarez C. Vigny A. Helle K. B. (1985). New dopaminergic terminal fields in the motor, visual (area 18b) and retrosplenial cortex in the young and adult rat. Immunocytochemical and catecholamine histochemical analyses. Neuroscience 15, 983–998. 10.1016/0306-4522(85)90248-9 2864660

[B3] Cheng J. Giguere P. M. Schmerberg C. M. Pogorelov V. M. Rodriguiz R. M. Huang X. P.. (2016). Further advances in optimizing (2-phenylcyclopropyl)methylamines as novel serotonin 2C agonists: effects on hyperlocomotion, prepulse inhibition, and cognition models. J. Med. Chem. 59, 578–591. 10.1021/acs.jmedchem.5b01153 26704965 PMC8317212

[B4] Clausen B. Schachtman T. R. Mark L. T. Reinholdt M. Christoffersen G. R. J. (2011). Impairments of exploration and memory after systemic or prelimbic D1-receptor antagonism in rats. Behav. Brain Res. 223, 241–254. 10.1016/j.bbr.2011.03.069 21497169

[B5] De Bundel D. Femenía T. Dupont C. M. Konradsson-Geuken A. Feltmann K. Schilström B.. (2013). Hippocampal and prefrontal dopamine D1/5 receptor involvement in the memory-enhancing effect of reboxetine. Int. J. Neuropsychopharmacol. 16, 2041–2051. 10.1017/S1461145713000370 23672849

[B6] de Landeta A. B. Pereyra M. Medina J. H. Katche C. (2020). Anterior retrosplenial cortex is required for long-term object recognition memory. Sci. Rep. 10, 1–13. 10.1038/s41598-020-60937-z 32152383 PMC7062718

[B7] de Landeta A. B. Pereyra M. Miranda M. Bekinschtein P. Medina J. H. Katche C. (2021). Functional connectivity of anterior retrosplenial cortex in object recognition memory. Neurobiol. Learn. Mem. 186:107544. 10.1016/j.nlm.2021.107544 34737148

[B8] de Lima M. N. M. Presti-Torres J. Dornelles A. Siciliani Scalco F. Roesler R. Garcia V. A.. (2011). Modulatory influence of dopamine receptors on consolidation of object recognition memory. Neurobiol. Learn. Mem. 95, 305–310. 10.1016/j.nlm.2010.12.007 21187154

[B9] Detrait E. R. Carr G. V. Weinberger D. R. Lamberty Y. (2016). Brain catechol-O-methyltransferase (COMT) inhibition by tolcapone counteracts recognition memory deficits in normal and chronic phencyclidine-treated rats and in COMT-Val transgenic mice. Behav. Pharmacol. 27, 415–421. 10.1097/FBP.0000000000000208 26919286 PMC4935608

[B10] Diop L. Gottberg E. Brière R. Grondin L. Reader T. A. (1988). Distribution of dopamine D1 receptors in rat cortical areas, neostriatum, olfactory bulb and hippocampus in relation to endogenous dopamine contents. Synapse 2, 395–405. 10.1002/syn.890020406 2973141

[B11] Furini C. R. G. Myskiw J. C. Schmidt B. E. Marcondes L. A. Izquierdo I. (2014). D1 and D5 dopamine receptors participate on the consolidation of two different memories. Behav. Brain Res. 271, 212–217. 10.1016/j.bbr.2014.06.027 24959860

[B12] Gonzalez M. C. Rossato J. I. Radiske A. Bevilaqua L. R. M. Cammarota M. (2021). Dopamine controls whether new declarative information updates reactivated memories through reconsolidation. Proc. Natl. Acad. Sci. U. S. A. 118, 3–5. 10.1073/pnas.2025275118 34253612 PMC8307459

[B13] Gumuslu E. Mutlu O. Sunnetci D. Ulak G. Celikyurt I. K. Cine N.. (2014). The antidepressant agomelatine improves memory deterioration and upregulates CREB and BDNF gene expression levels in unpredictable chronic mild stress (UCMS)-exposed mice. Drug Target Insights 8, 11–21. 10.4137/DTI.S13870 24634580 PMC3948735

[B14] Guzmán-Ramos K. Moreno-Castilla P. Castro-Cruz M. McGaugh J. L. Martínez-Coria H. LaFerla F. M.. (2012). Restoration of dopamine release deficits during object recognition memory acquisition attenuates cognitive impairment in a triple transgenic mice model of Alzheimer's disease. Learn. Mem. 19, 453–460. 10.1101/lm.026070.112 22984283

[B15] Katche C. Dorman G. Gonzalez C. Kramar C. P. Slipczuk L. Rossato J. I.. (2013). On the role of retrosplenial cortex in long-lasting memory storage. Hippocampus 23, 295–302. 10.1002/hipo.22092 23355414

[B16] Lima K. R. de Souza da Rosa A. C. Picua S. S. Souza e Silva S. Soares N. M. Mello-Carpes P. M. (2022). Novelty promotes recognition memory persistence by D1 dopamine receptor and protein kinase A signalling in rat hippocampus. Eur. J. Neurosci. 55, 78–90. 10.1111/ejn.15568 34904283

[B17] Lisman J. E. Grace A. A. (2005). The hippocampal-VTA loop: controlling the entry of information into long-term memory. Neuron 46, 703–713. 10.1016/j.neuron.2005.05.002 15924857

[B18] Millan M. J. Gobert A. Lejeune F. Dekeyne A. Newman-Tancredi A. Pasteau V.. (2003). The novel melatonin agonist agomelatine (S20098) is an antagonist at 5-hydroxytryptamine2C receptors, blockade of which enhances the activity of frontocortical dopaminergic and adrenergic pathways. J. Pharmacol. Exp. Ther. 306, 954–964. 10.1124/jpet.103.051797 12750432

[B19] Millan M. J. Newman-Tancredi A. Quentric Y. Cussac D. (2001). The “selective” dopamine D1 receptor antagonist, SCH23390, is a potent and high efficacy agonist at cloned human serotonin2C receptors. Psychopharmacology 156, 58–62. 10.1007/s002130100742 11465634

[B20] Morales M. Margolis E. B. (2017). Ventral tegmental area: cellular heterogeneity, connectivity and behaviour. Nat. Rev. Neurosci. 18, 73–85. 10.1038/nrn.2016.165 28053327

[B21] Nagai T. Takuma K. Kamei H. Ito Y. Nakamichi N. Ibi D.. (2007). Dopamine D1 receptors regulate protein synthesis-dependent long-term recognition memory via extracellular signal-regulated kinase 1/2 in the prefrontal cortex. Learn. Mem. 14, 117–125. 10.1101/lm.461407 17337702 PMC1838552

[B22] Neves B. H. S. Barbosa G. P. D. R. de Souza da Rosa A. C. Picua S. S. Gomes G. M. Sosa P. M.. (2020). On the role of the dopaminergic system in the memory deficits induced by maternal deprivation. Neurobiol. Learn. Mem. 173. 10.1016/j.nlm.2020.107272 32622955

[B23] Oades R. D. Halliday G. M. (1987). Ventral tegmental (A10) system: neurobiology. 1. Anatomy and connectivity. Brain Res. Rev. 12, 117–165. 10.1016/0165-0173(87)90011-7 3107759

[B24] Paxinos G. Watson C. (2007). The Rat Brain in Stereotaxic Coordinates, Vol. 170, 6th Edn. London: Elsevier Academic Press, 547–612.

[B25] Pitsikas N. Sakellaridis N. (2005). The 5-HT2C receptor antagonist RO 60-0491 counteracts rats' retention deficits in a recognition memory task. Brain Res. 1054, 200–202. 10.1016/j.brainres.2005.06.057 16040008

[B26] Rossato J. I. Köhler C. A. Radiske A. Lima R. H. Bevilaqua L. R. M. Cammarota M. (2015). State-dependent effect of dopamine D1/D5 receptors inactivation on memory destabilization and reconsolidation. Behav. Brain Res. 285, 194–199. 10.1016/j.bbr.2014.09.009 25219363

[B27] Rossato J. I. Radiske A. Kohler C. A. Gonzalez C. Bevilaqua L. R. Medina J. H.. (2013). Consolidation of object recognition memory requires simultaneous activation of dopamine D1/D5 receptors in the amygdala and medial prefrontal cortex but not in the hippocampus. Neurobiol. Learn. Mem. 106, 66–70. 10.1016/j.nlm.2013.07.012 23891712

[B28] Savalli G. Bashir Z. I. Warburton E. C. (2015). Regionally selective requirement for D1/D5 dopaminergic neurotransmission in the medial prefrontal cortex in object-in-place associative recognition memory. Learn. Mem. 22, 69–73. 10.1101/lm.036921.114 25593292 PMC4341361

[B29] Vargas L. S. Ramires Lima K. Piaia Ramborger B. Roehrs R. Izquierdo I. Mello-Carpes P. B. (2020). Catecholaminergic hippocampal activation is necessary for object recognition memory persistence induced by one-single physical exercise session. Behav. Brain Res. 379. 10.1016/j.bbr.2019.112356 31730785

